# Life at high salt concentrations, intracellular KCl concentrations, and acidic proteomes

**DOI:** 10.3389/fmicb.2013.00315

**Published:** 2013-11-05

**Authors:** Aharon Oren

**Affiliations:** Department of Plant and Environmental Sciences, The Alexander Silberman Institute of Life Sciences, The Hebrew University of JerusalemJerusalem, Israel

**Keywords:** acidic proteins, osmotic adaptation, halophilic, marine bacteria, anaerobic, *Halanaerobiaceae*

## Abstract

Extremely halophilic microorganisms that accumulate KCl for osmotic balance (the *Halobacteriaceae*, *Salinibacter*) have a large excess of acidic amino acids in their proteins. This minireview explores the occurrence of acidic proteomes in halophiles of different physiology and phylogenetic affiliation. For fermentative bacteria of the order *Halanaerobiales*, known to accumulate KCl, an acidic proteome was predicted. However, this is not confirmed by genome analysis. The reported excess of acidic amino acids is due to a high content of Gln and Asn, which yield Glu and Asp upon acid hydrolysis. The closely related *Halorhodospira halophila* and *Halorhodospira halochloris* use different strategies to cope with high salt. The first has an acidic proteome and accumulates high KCl concentrations at high salt concentrations; the second does not accumulate KCl and lacks an acidic proteome. Acidic proteomes can be predicted from the genomes of some moderately halophilic aerobes that accumulate organic osmotic solutes (*Halomonas elongata*, *Chromohalobacter salexigens*) and some marine bacteria. Based on the information on cultured species it is possible to understand the pI profiles predicted from metagenomic data from hypersaline environments.

## INTRODUCTION

In a study of the proteins of *Halobacterium* and* Halococcus,* Reistad (1970) noted an unusual amino acids composition of the cells’ bulk protein: a great excess of the acidic amino acids glutamate and aspartate compared to the basic amino acids lysine and arginine. Analysis of the genome of *Halobacterium *NRC-1 (Ng et al., 2000) and related organisms (Oren, 2013a) has confirmed the special properties of the proteins of this group of Archaea. The acidic proteins of the *Halobacteriaceae* typically require high salt concentrations for structural stability and activity, and the presence of such an acidic proteome was considered to be correlated with the accumulation of molar concentrations of KCl to provide osmotic balance to the cells (Lanyi, 1974; Mevarech et al., 2000; Oren, 2013b).

A different strategy of osmotic adaptation in halophilic and halotolerant microorganisms is the accumulation of organic osmotic solutes (glycine betaine, ectoine, glycerol, simple sugars, etc., often termed “compatible solutes”). Such molecules are generally uncharged or zwitterionic, and their presence in high intracellular concentrations does not require far-going adaptation of the proteins. The intracellular solute concentrations can be rapidly adjusted according to the outside salinity, so that microorganisms using this “low-salt-in” strategy can often adapt to life at a wide range of salinities (Galinski, 1995; Oren, 2002a,b, 2011, 2013b).

Early attempts to correlate the mode of osmotic adaptation used by halophilic microorganisms with their phylogenetic position yielded a relatively simple picture: the “high-salt-in” strategy was thought to be limited to the *Halobacteriaceae* within the Archaea domain. Among the Bacteria a single group was known that had high intracellular KCl concentrations and allegedly had a highly acidic proteome: the anaerobic fermentative *Halanaerobiales* (*Firmicutes*). Other groups of Bacteria use organic osmotic solutes, generally in a pattern correlated with their phylogenetic affiliation; organic solutes are also found in halophilic methanogens (Archaea) and in salt-adapted eukaryotic microorganisms (Oren, 2008; Trüper et al., 1991). This represented the state of our knowledge up to turn of the century. Since then the relatively simple picture got complicated by new information and insights. Some of these new data are discussed below.

## *Salinibacter ruber*, ITS MODE OF OSMOTIC ADAPTATION AND THE PROPERTIES OF ITS PROTEINS

*Salinibacter ruber* (*Bacteroidetes*) is a red-pigmented aerobic heterotrophic extremely halophile, first isolated from Spanish saltern crystallizer ponds (Antón et al., 2002), but now known to be distributed worldwide in neutral-pH water bodies at or near salt saturation. This interesting organism shares many key properties with the *Halobacteriaceae* with which it shares its habitat (Oren, 2013c). These include the accumulation of molar concentrations of KCl intracellularly, insignificant concentrations of organic osmotic solutes (Oren et al., 2002), a highly acidic nature of the bulk protein, and a strict salt requirement of key enzymes (Oren and Mana, 2002). High intracellular KCl concentrations were also measured in the phylogenetically related *Salisaeta longa* (Vaisman and Oren, 2009; Vaisman and Oren, unpublished results).

**FIGURE 1:**
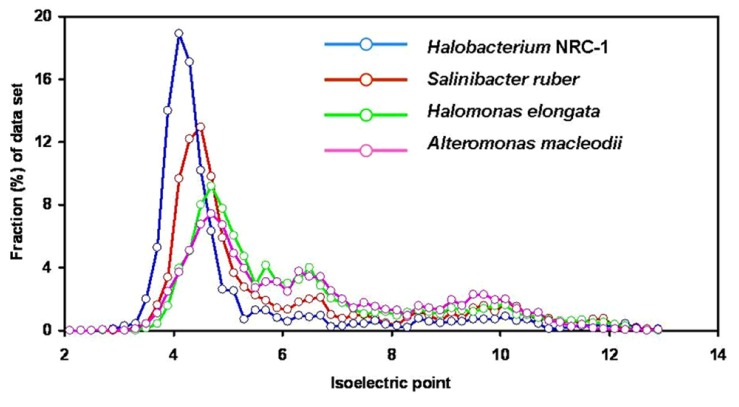
**Isoelectric point profiles, calculated at 0.2 intervals, of predicted proteins encoded by the genomes of Halobacterium NRC-1 (2,675 proteins; median pI 5.03), the extreme halophilic bacterium *Salinibacter ruber* (2,845 proteins; median pI 5.92), the aerobic moderately halophilic bacterium *Halomonas elongata* (3,474 proteins; median pI 6.32) and the aerobic marine bacterium *Alteromonas macleodii* (4,396 proteins; median pI 6.46).** From the genome annotations (Ng et al., 2000; Mongodin et al., 2005; Schwibbert et al., 2011) sequences encoding proteins or putative proteins were extracted, and for each protein sequence the predicted the pI value was calculated using the programs in the Galaxy platform (; Giardine et al., 2005; Blankenberg et al., 2010; Goecks et al., 2010). With kind permission from Springer Science+Business Media: Extremophiles, Elevi Bardavid and Oren (2012b), compiled from data presented in Figures 1–3.

Analysis of the *Salinibacter* genome (Mongodin et al., 2005) confirmed the highly acidic nature of most of its proteins. The median pI value of 5.92 for the proteins encoded by the *S. ruber* genome is slightly higher than that for *Halobacterium* NRC-1 (5.03; **Figure 1**). *Salinibacter* can be considered as an example of convergent evolution mediated by extensive gene exchange with archaeal halophiles found in the same habitat. The combination of the “salt-in” strategy and the possession of salt-dependent, highly acidic proteins is thus not necessarily limited to the *Halobacteriaceae* lineage of aerobic halophilic Archaea.

## THE NATURE OF THE PROTEOME OF THE *Halanaerobiales* AND OTHER HALOPHILIC ANAEROBES WITHIN THE BACTERIAL DOMAIN

The order *Halanaerobiales*, families *Halanaerobiaceae* and *Halobacteroidaceae* (Rainey et al., 1995) forms a phylogenetically coherent group of anaerobic bacteria affiliated with the low G+C *Firmicutes* (Kivistö and Karp, 2011; Oren, 2013d). Members of the group have been found in sediments of Great Salt Lake, Utah, the Dead Sea, salterns, oil wells, and in alkaline hypersaline lakes (Lake Magadi, Kenya, Big Soda Lake, Nevada). Most species grow optimally at 10–15% NaCl, and some tolerate salt up to saturation. Most ferment sugars to acetate, ethanol, H_2_, CO_2_, and other products. One of these (*Halothermothrix orenii*, isolated from a warm saline lake in Tunisia), is a true thermophilic (up to 68°C; optimum at 60°C) halophile (growth up to 20% NaCl; Cayol et al., 1994). Some genera (*Acetohalobium*, *Natroniella*) have a homoacetogenic metabolism. *Selenihalanaerobacter shriftii* grows by anaerobic respiration with selenate or nitrate as electron acceptor.

Examination of the cytoplasm of members of the *Halanaerobiales* did not show significant concentrations of organic osmotic solutes (Oren, 1986; Rengpipat et al., 1988), with the possible exception of the finding of glycine betaine in *Orenia salinaria* grown in media containing yeast extract (Mouné et al., 2000). However, high ionic concentrations (K^+^, Na^+^, Cl^-^), sufficient to provide osmotic balance, were measured in *Halanaerobium praevalens* (Oren, 1986; Oren et al., 1997), *Halanaerobium acetethylicum* (Rengpipat et al., 1988), *Halobacteroides halobius* (Oren, 1986), and *Natroniella acetigena *(Detkova and Pusheva, 2006). Studies performed on glyceraldehyde-3-phosphate dehydrogenase, NAD-linked alcohol dehydrogenase, pyruvate dehydrogenase, and methyl viologen-linked hydrogenase from *H. acetethylicum* (Rengpipat et al., 1988), carbon monoxide dehydrogenase of *N. acetigena* (Detkova and Boltyanskaya, 2006; Detkova and Pusheva, 2006), the fatty acid synthetase complex of *H. praevalens* (Oren and Gurevich, 1993), and hydrogenase and carbon monoxide dehydrogenase of *Acetohalobium arabaticum* (Zavarzin et al., 1994) showed that all these enzymes function well in the presence of molar salt concentrations, and many need high salt for optimal activity. Therefore the “high-salt-in” strategy was assumed to be the mode of osmotic adaptation in this group (Kivistö and Karp, 2011; Oren, 2013d).

Based on these observations the proteome of the members of the *Halanaerobiales* was predicted to have a strongly acidic nature. Indeed, analysis of acid hydrolysates of *Halanaerobium* *praevalens*, *Halanaerobium saccharolyticum*, *Natroniella acetigena*, *Halobacteroides halobius*, and *Sporohalobacter lortetii* suggested that the bulk protein of all these species may have a strongly acidic nature (Oren, 1986; Detkova and Boltyanskaya, 2006). However, it must be remembered that during acid hydrolysis, the neutral asparagine and glutamine are deaminated to form aspartate and glutamate.

**FIGURE 2:**
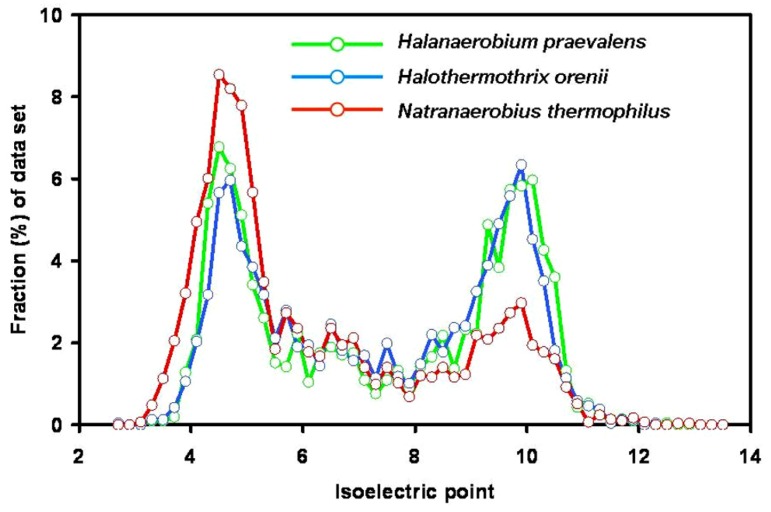
**Isoelectric point profiles, calculated at 0.2 intervals, of predicted proteins encoded by the genomes of the three fermentative halophilic anaerobes: *Halanaerobium praevalens* GSL^**T**^ (2,110 proteins; median pI 7.42), the thermophilic *Halothermothrix orenii *H168^**T**^ (2,365 proteins, median pI 7.41) and the alkaliphilic and thermophilic *Natranaerobius thermophilus* JW.NM-WN-LF^**T**^ (2,906 proteins; median pI 6.27).** From the genome annotations (Mavromatis et al., 2009; Ivanova et al., 2011; Zhao et al., 2011) sequences encoding proteins or putative proteins were extracted, and pI profiles were calculated as for **Figure 1**. With kind permission from Springer Science+Business Media: Extremophiles, compiled from data presented in Elevi Bardavid and Oren (2012a) – **Figure 1**, and Elevi Bardavid and Oren (2012b) – **Figure 4**.

The first evidence against a highly acidic proteome in members of the *Halanaerobiales* was published in 1987 when it was shown that the *H. praevalens* ribosomal A-protein is not particularly rich in acidic amino acids (Matheson et al., 1987). Today genome sequences of three members of the group are available: *H. praevalens *GSL^T^ (Ivanova et al., 2011), a haloalkaliphilic strain from Soap Lake, WA, USA, described as “*Halanaerobium hydrogeniformans*” (Brown et al., 2011), and *Halothermothrix orenii *H168^T^ (Mavromatis et al., 2009). Analysis of these three genomes did not show preferential use of acidic amino acids and no low content of basic amino acids (Elevi Bardavid and Oren, 2012a; **Figure 2**). It was earlier suggested that the proteins of *H. orenii* may lack a pronounced acidic nature as a special adaptation toward growth at high temperatures (Mijts and Patel, 2001; Mavromatis et al., 2009). The properties of the other two genomes show that also the mesophilic species lack an acidic proteome. The bimodal distribution of the pI values with peaks around 4.6–4.8 and 9.8–10.2 is similar to that of the non-halophiles *Bacteroides fragilis* and *Chlorobaculum tepidum* (Mongodin et al., 2005). The main reason for the apparent discrepancy between the bulk protein analyses, showing a pronounced acid nature, and the analysis of the proteins encoded by the genomes, is the high content of glutamine and asparagine, which lose their amide group during the acid hydrolysis procedure involved in sample preparation for amino acid analysis.

Still there is no reason to doubt the presence of high ionic concentrations within the cytoplasm to balance the osmotic pressure of the medium. Analysis of the three *Halanaerobiales* genomes did not show clear evidence for pathways leading to the synthesis of organic osmotic solutes. A gene for sucrose phosphate synthase was identified in *H. orenii*, which may point to the possibility of sucrose biosynthesis (Mavromatis et al., 2009). Whether indeed sucrose is present in the cells at high concentrations, remains to be ascertained. The possibility must be taken into account that the anaerobic halophilic of the *Halanaerobiales* group use a “high-salt-in” strategy of osmotic adaptation but have not adopted the pattern of acidic, low-pI proteins commonly associated with haloadaptation in the aerobic halophiles (*Halobacteriaceae*, *Salinibacter*). A renewed study of the special properties of the *Halanaerobiales* may therefore provide new insights into the strategies available to the prokaryote world to thrive at high salt concentrations (Elevi Bardavid and Oren, 2012a).

The genomes of two anaerobic fermentative halophiles belonging to other phylogenetic lineages were recently sequenced. One is *Flexistipes sinusarabici* MAS 10^T^, isolated from a deep-sea brine pool on the bottom of the Red Sea (Fiala et al., 1990; Lapidus et al., 2011). It was classified as a member of the *Deferribacteres*, a deep branch within the Bacteria; it grows between 3 and 10% salt and possibly higher. Its mode of osmotic adaptation is yet unknown. Its pI profile (bimodal, with a median pI of 7.47) resembles that of *Halanaerobium*. The second is *Natranaerobius thermophilus* JW.NM-WN-LF^T^ an anaerobic halothermoalkaliphile isolated from the Wadi An Natrun lakes in Egypt. It was classified in the newly established order *Natranaerobiales* (*Clostridia*), requires 3.1–4.9 M Na^+^, and is markedly thermophilic (optimum growth at 53°C) and alkaliphilic (optimum pH 9.5; Mesbah et al., 2007). Analysis of its genome (Zhao et al., 2011) yielded a markedly acidic proteome (median pI 6.27; Elevi Bardavid and Oren, 2012b; **Figure 2**). Comparison of the proteins of five anaerobic halophiles of different phylogenetic lineages and with different temperature and pH optima thus shows great variations in the acidic nature of the proteome.

## DISPARATE OSMOTIC ADAPTATION STRATEGIES WITHIN THE GENUS *Halorhodospira*

The genus *Halorhodospira* currently contains four species: the type species *H. halophila*, *H. neutriphila*, *H. halochloris* and *H. abdelmalekii*. With respect to salt requirement and tolerance they are quite similar, and all tolerate NaCl at concentrations up to 25% or higher. They can be divided into two groups, phylogenetically separated on the basis of 16S rRNA gene sequences: *H. halophila* and *H. neutriphila* contain bacteriochlorophyll *a* and carotenoids of the spirilloxanthin group, while *H. halochloris* and *H. abdelmalekii* contain bacteriochlorophyll *b *and rhopdopin carotenoids (Imhoff and Süling, 1996; Hirschler-Réa et al., 2003; Oren, 2013e). With respect to their mode of osmotic adaptation they were always considered to be a prime example of organisms that use organic compatible solutes. *H. halophila*, *H. halochloris* and *H. abdelmalekii* were all shown to produce glycine betaine as osmotic solute, with minor amounts of ectoine and trehalose (Trüper et al., 1991). Ectoine, now known to be the most widespread osmotic solute in the prokaryote world, was first discovered in *H. halochloris *(Galinski et al., 1985).

In view of their common phylogeny and documented content of organic osmotic solutes, the finding of an acidic proteome and of high intracellular KCl concentrations in *H. halophila* but not in *H. halochloris* (Deole et al., 2013) came as a big surprise. While the latter does not accumulate KCl, the first contains high KCl when grown at high salt (35%) but not at low salt (5%). The genus *Halorhodospira* thus presents a thus far unique case in which different combinations of KCl concentrations, production of organic osmotic solutes, and presence of acidic vs. non-acidic proteomes are used for osmotic adaptation in phylogenetically closely related species. The authors concluded that “proteome acidity is not driven by stabilizing interactions between K^+^ ions and acidic side chains but by the need for maintaining sufficient solvation and hydration of the protein surface at high salinity …,” and they proposed that “obligate protein halophilicity is a non-adaptive property resulting from genetic drift in which constructive neutral evolution progressively incorporates weak K^+^-binding sites on an increasingly acidic protein surface” (Deole et al., 2013).

## ACIDIC PROTEOMES IN MODERATELY HALOPHILIC *Gammaproteobacteria*

There is no *a priori *reason to assume that moderately halophilic aerobic heterotrophic bacteria that synthesize and/or accumulate organic compatible solutes should have a high acidic proteome adapted to function in the presence of high intracellular salt concentrations. A first survey of the proteins of the gammaproteobacterium *Chromohalobacter salexigens* DSM 3043^T^, based on 238 out of the 3,319 proteins encoded by its genome, indeed showed that most selected proteins were no more acidic than comparable proteins from non-halophilic counterparts. A notable exception was found for periplasmic proteins exposed to the high medium salinity (Oren et al., 2005).

Analysis of the entire *C. salexigens* genome, together with that of the phylogenetically related moderate halophile *Halomonas elongata* 1H9^T^ (Schwibbert et al., 2011) showed large peaks of acidic proteins (maximum at pI 4.4–5.0 and 4.5–5.1, respectively) in the pI profiles of the predicted proteins. The median pI values for the proteins encoded by these genomes are 6.60 and 6.32, respectively (Elevi Bardavid and Oren, 2012b). These values are still in the low pI range, albeit somewhat higher than those reported for “high-salt-in” organisms such as *Halobacterium* and *Salinibacter* (**Figure 2**). Both organisms synthesize ectoine as compatible solute and accumulate glycine betaine when available in the medium.

Such acidic proteomes are found not only in halophilic and highly halotolerant members of the *Gammaproteobacteria*, but also in typically marine members of the group. Analysis of the pI distribution of the proteins predicted from the genomes of *Alteromonas macleodii* ATCC 27126^T^ (Ivars-Martínez et al., 2008), a representative of a genus ubiquitous in the world’s oceans, and of the luminescent *Aliivibrio fischeri* strain MJ11 (Mandel et al., 2009) showed a pronounced peak in the acidic range (maximum at pI values of 4.6–4.8), with median pI values of 6.46 and 6.52, respectively (Elevi Bardavid and Oren, 2012b).

## ACIDIC METAPROTEOMES IN HYPERSALINE ENVIRONMENTS

Metagenomic data from saline and hypersaline environments can be subjected to analyses similar to those shown above for microbial isolates. As shown by Rhodes et al. (2010), there is a general trend of increased average protein acidity (as expressed by the ratio of acidic to basic amino acids) with increased salinity. The highest salinity environments (the Dead Sea, saltern crystallizer ponds) have the greatest excess of acidic amino acids in the proteins encoded by the recovered DNA ([Glu + Asp]/[Lys + Arg + His] = 1.42–1.26). This could be expected as high-salt-in strategists (species of *Halobacteriaceae*, *Salinibacter*) with highly acidic proteomes dominate their biota. Metagenomes from different samples from the marine environment gave values in the range 0.86–0.95, and the benthic microbial mats in the 9% salt lagoons of Guerrero Negro, Mexico (Kunin et al., 2008), yielded an intermediate value of 1.01 on the average.

The finding that the pI distribution of the proteins encoded by the metagenome of the Guerrero Negro microbial mats showed an acid-shifted proteome (major peak at pI 4.5–4.9, median pI 6.8) as compared to non-halophilic or marine environments was at first puzzling, as at that salinity microorganisms are expected to use organic osmotic solutes, without the need to adapt their proteins to high salt. Kunin et al. (2008) concluded that the enhanced acidic nature of the proteins is linked to the increased salinity, explained as an example of species-independent molecular convergence in a microbial community. However, as documented above, many moderate halophiles, also those that do not accumulate organic osmotic solutes, show a broad peak at pI values 4.5–4.9 (Elevi Bardavid and Oren, 2012b). In comparison to the proteins encoded by the genomes of moderately halophilic aerobes and even certain marine bacteria (**Figure 2**), the metaproteome encoded by the metagenome of the different layers within the 9% salt Guerrero Negro microbial mats is not conspicuously acid-shifted.

The finding that marine metagenomes do not encode for metaproteomes enriched in acidic proteins (Rhodes et al., 2010) needs to be evaluated in view of the above-mentioned observation that some typically marine *Gammaproteobacteria* (*Alteromonas* and *Vibrio* spp.) are rich in low-pI proteins. A possible explanation is that other, possibly very abundant marine bacteria show the opposite trend. The small genome of “Pelagibacter ubique” (*Alphaproteobacteria*; the “SAR-11” phylotype; Giovannoni et al., 2005) encodes for 1,393 proteins with an overall excess of 2% (Lys + Arg) – (Glu + Asp). For comparison, *Halobacterium*, *Salinibacter* and *H. elongata* and *Halomonas *all show an excess of acidic amino acids (7.5, 4.1, and 2.8 mol %, respectively; Elevi Bardavid and Oren, 2012b). Most “Pelagibacter” proteins have pI values between 9.4 and 10.8, with a median value of 8.42.

## FINAL COMMENTS

The genomic and metagenomic data discussed above show that dominance of acidic proteins in halophilic microorganisms is by no means restricted to the *Halobacteriaceae* and to *Salinibacter* which resembles the *Halobacteriaceae* in many properties. Somewhat less acidic proteomes are found in many moderately halophilic and even in some marine bacteria, organisms that exclude salt from their cytoplasm to a large extent (Elevi Bardavid and Oren, 2012b). On the other hand, the analysis of the genomes of different anaerobic halophiles (*Halanaerobiales* and others) unexpectedly failed to show a highly acidic proteome (Elevi Bardavid and Oren, 2012a). The case of the two *Halorhodospira* species demonstrates that phylogenetically very closely related organisms may use completely different strategies for osmotic adaptation, and accordingly have highly different amino acid signatures of their proteins. The more or less coherent picture of a clear correlation between phylogenetic affiliation and modes of salt adaptation that was apparent in the past (Trüper et al., 1991; Oren, 2008) needs therefore drastic revision. We must rethink our concepts about the correlation between acidic proteomes, salt requirement and tolerance, accumulation of KCl, use of organic osmotic solutes, and microbial phylogeny and taxonomy.

A recently published analysis of the structure of primitive proteins that may have been formed from “prebiotic” amino acids expected to have been available at the time life originated on Earth showed that the predicted foldable proteins have a substantial acidification of pI and possessed halophilic properties (Longo et al., 2013). The question whether the environment for primordial life may have been hypersaline has been addressed earlier (Dundas, 1998). Therefore the issues discussed above may even have direct implications for our ideas on the origin of life and the properties of the earliest organisms that inhabited our planet.

## Conflict of Interest Statement

The authors declare that the research was conducted in the absence of any commercial or financial relationships that could be construed as a potential conflict of interest.
